# A Hydrophilic Interaction Liquid Chromatography–Tandem Mass Spectrometry Quantitative Method for Determination of Baricitinib in Plasma, and Its Application in a Pharmacokinetic Study in Rats

**DOI:** 10.3390/molecules25071600

**Published:** 2020-03-31

**Authors:** Essam Ezzeldin, Muzaffar Iqbal, Yousif A. Asiri, Azza A Ali, Prawez Alam, Toqa El-Nahhas

**Affiliations:** 1 Department of Pharmaceutical Chemistry and Drug Bioavailability Unit, Central Laboratory, College of Pharmacy, King Saud University, Riyadh 11451, Saudi Arabia or muziqbal@ksu.edu.sa (M.I.); 2 National Organization for Drug Control and Research, Cairo 12611, Egypt; 3 Clinical Pharmacy Department, College of Pharmacy, King Saud University, Riyadh 11451, Saudi Arabia; yasiri@ksu.edu.sa; 4 Pharmacology and Toxicology Department, Faculty of Pharmacy (Girls) Al-Azhar University, Cairo 11754, Egypt; azzamro@gmail.com; 5 Pharmacognosy Department, College of Pharmacy, Prince Sattam Bin Abdulaziz University, Al-Kharj 11942, Saudi Arabia; p.alam@psau.edu.sa

**Keywords:** baricitinib, UPLC-MS/MS, pharmacokinetic study, irbersartan

## Abstract

Baricitinib, is a selective and reversible Janus kinase inhibitor, is commonly used to treat adult patients with moderately to severely active rheumatoid arthritis (RA). A fast, reproducible and sensitive method of liquid chromatography-tandem mass spectrometry (LC-MS/MS) for the quantification of baricitinib in rat plasma has been developed. Irbersartan was used as the internal standard (IS). Baracitinib and IS were extracted from plasma by liquid–liquid extraction using a mixture of n-hexane and dichloromethane (1:1) as extracting agent. Chromatographic separation was performed using Acquity UPLC HILIC BEH 1.7 µm 2.1 × 50 mm column with the mobile phase consisting of 0.1% formic acid in acetonitrile and 20 mM ammonium acetate (pH 3) (97:3). The electrospray ionization in the positive-mode was used for sample ionization in the multiple reaction monitoring mode. Baricitinib and the IS were quantified using precursor-to-production transitions of *m*/*z* 372.15 > 251.24 and 429.69 > 207.35 for baricitinib and IS, respectively. The method was validated according to the recent FDA and EMA guidelines for bioanalytical method validation. The lower limit of quantification was 0.2 ng/mL, whereas the intra-day and inter-day accuracies of quality control (QCs) samples were ranged between 85.31% to 89.97% and 87.50% to 88.33%, respectively. Linearity, recovery, precision, and stability parameters were found to be within the acceptable range. The method was applied successfully applied in pilot pharmacokinetic studies.

## 1. Introduction

Rheumatoid arthritis (RA) is a progressive and long-lasting autoimmune disorder that most commonly affects the joints. RA is a chronic systemic inflammatory disorder which affects 0.5–1% of the adult population. It is characterized by persistent inflammation of synovial joints resulting in a symmetrical inflammatory polyarthritis with progressive erosive destruction of joints including cartilage and bone deformity and damage [1]. The main symptoms of RA are joint pain, inflammation, swelling, stiffness and generalized fatigue with episodes of RA flares and remissions. RA is a systemic disease that can have a significant effect on other organs, including eyes [2], heart [3,4], and lung [5].

**Figure 1:**
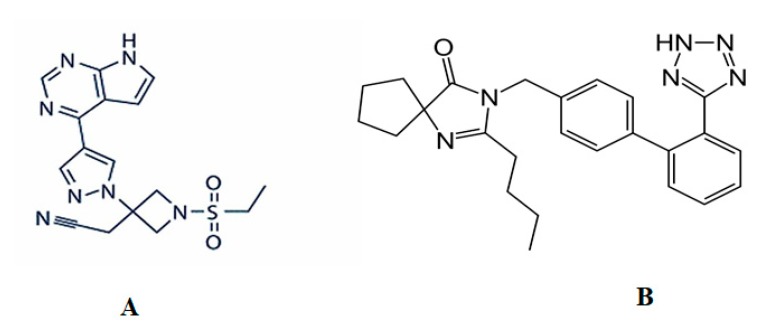
Chemical structures of baricitinib (**A**) and irbesartan (**B**).

Janus kinase inhibitors are anti-rheumatic drugs that target the intracellular kinase (JAK). JAKs play an important role in the signaling of many cytokines and hematopoietic growth and development [6]. Baricitinib (Figure 1A), a selective and reversible JAK1 and JAK2 inhibitor, is commonly used to treat moderate to severe active RA in adult patients with poor response to one or more tumor necrosis factor inhibitors (TNFis) [7]. Baricitinib has showed dose-dependent efficacy for up to 24 weeks with significant improvements in the signs and symptoms of RA in patients [8]. It has been reported that patients treated with baricitinib demonstrated consistent pain relief irrespective of the degree of inflammation control. Baricitinib treatment is associated with improvements in RA disease control, provides rapid pain relief, improves work productivity and is cost-effective in comparison to other RA drugs [9]. In addition, patients with hereditary immunodeficiency syndrome showed a complete disappearance of rheumatoid nodules following 12 months of baricitinib treatment. Moreover, baricitinib had a significant improvement in the signs and symptoms of systemic lupus erythematosus [10].

Baricitinib is rapidly absorbed after oral administration and reaches peak plasma concentrations at approximately 1 h. Baricitinib oral bioavailability is 79% and it is moderately bound (about 50%) to plasma proteins mainly albumin [11]. Baricitinib is primarily metabolized by CYP3A4 enzyme and it is a substrate of P-glycoprotein (Pgp), organic anionic transporter (OAT) 3, multidrug and toxic extrusion protein (MATE) 2-K and breast cancer resistance protein (BCRP). Baricitinib pharmacokinetics was found to be affected by food and concurrent administration of other drugs. The therapeutic effects and safety of baricitinib are related to the rate and extent of absorption [7]. It was also found that probenecid elevates the extent of baricitinb absorption, which represent in the potentiation of AUC_0-inf_ by twofold and decreased the renal clearance to 69%, while the rate of absorption (C_max_) was not significantly increased [12]. Furthermore, rifampicin, a potent CYP3A inducer, decreased baricitinib AUC without affecting C_max_ [13]. No clinically significant drug-interaction has been reported between CYP substrates, inhibitors, and inducers with baricitinib. Ketoconazole (CYP3A inhibitor) and fluconazole (CYP2C19/CYP2C9/CYP3A inhibitor) did not significantly affect baricitinib pharmacokinetics, however, baricitinib clearance was reduced by an inhibitor of the transporter, OAT3. Although co-administration of high-fat meal had no effect on baricitinib AUC, it reduced C_max_ by about 29% with 3 h delayed in t_max_ and had no clinical influence [14].

Baricitinib safety is a controversial area. Some studies showed that baricitinib was safe and well tolerated following treatment of patients with moderate to severe RA for 24 weeks [8,15,16]. However, other studies demonstrated that baricitinib induces a stable dose-response increase in LDL-C and HDL-C levels with developing the risk of thrombosis [17], elevates infection risk, particularly for herpes zoster [18] and GI perforations [19]. Substantial data concerning baricitinib embryotoxicity and teratogenicity in humans are lacking. However, pre-clinical animal studies revealed its embryotoxicity, teratogenicity, and adverse effect on bone development in utero at higher dosages [20].

RA therapy includes antirheumatic drugs, JAK inhibitors, biologic (such as tumor necrosis factor-α or and interleukin 6 inhibitors), and nonsteroidal anti-inflammatory drugs mostly in combinations [21]. Therefore, it is important to study baricitinib pharmacokinetics in different therapeutic approaches. Currently, TKIs including baricitinib are mostly used as a fixed dosage therapy with no consideration of inter-individual differences. Although this might be a practical tool, optimal target therapeutic plasma concentration can be achieved based on dosage individualization strategy using therapeutic drug monitoring to obtain rapid and effective clinical response with minimum incidence of adverse effects [21].

There are very limited resources for validated analytical methods of baricitinib in biological fluids. Both available methods measure baricitinib in combination with other drugs using either low purification technique, protein precipitation with low sensitivity and long run time [22] or solid phase extraction which is an expensive technique and required high volume of samples [23]. Hence, development of a fast and sensitive method for analysis of baricitinib in biological fluids is of great importance for better understanding of baricitinib performance in both routine therapeutic drug monitoring and clinical trials. The present study aimed to develop a new, rapid, sensitive, reproducible and cost-effective method for determination of baricitinib in plasma and to study its applicability in a pharmacokinetic study in rats.

## 2. Results and Discussion

### 2.1. Chromatography and Mass Spectrometry Conditions 

**Figure 2:**
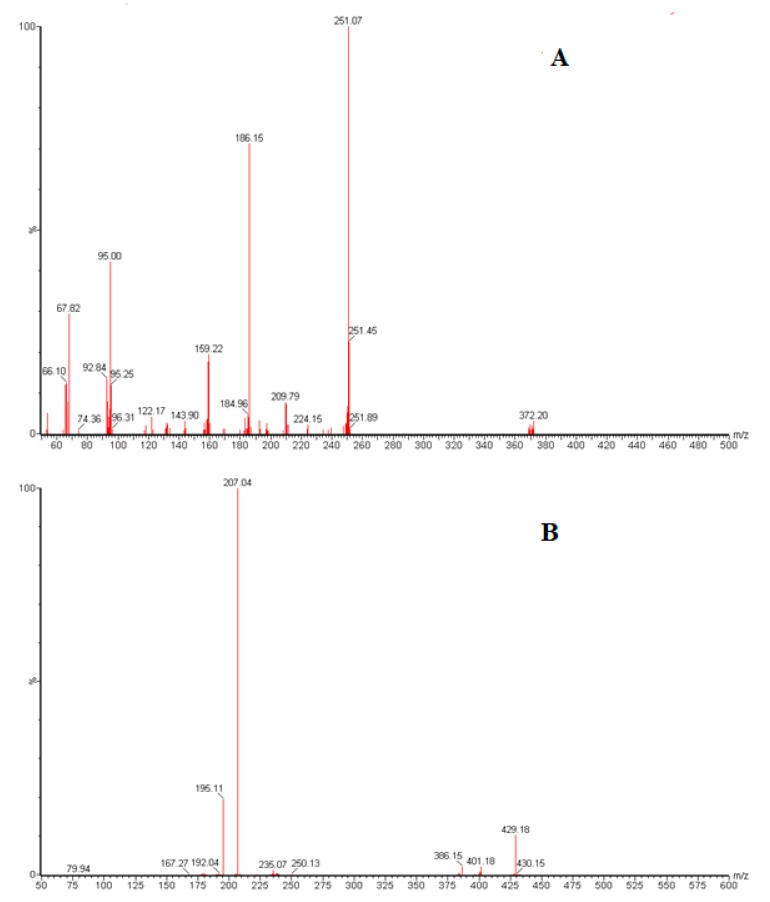
Positive ESI product ion mass spectra of baricitinib (**A**) and irbersartan (**B**) obtained from collisional activated dissociation of the precursor ion *m*/*z* 372.20 and 429.18, respectively.

Mass spectrometry parameters were optimized to achieve better ionization of baricitinib and IS molecules. MS optimization was obtained by direct injection of baricitinib and IS into the mass spectrometer. The mass spectra revealed that more stable and higher responses were obtained in positive-ion mode. Cone voltage and collision energy parameters were also optimized. The mass spectra revealed peaks for the most abundant protonated molecular ions [M + H]^+^ for baricitinib and IS were obtained at *m*/*z* 372.36 and 429.69, respectively. The major (predominant) fragment ions observed in each product spectrum were at *m*/*z* 251.24 and 207.15 for baricitinib and IS, respectively (Figure 2). Irbersartan was used as internal standard as it has some physical properties similar to the anayte such as poor solubility in buffer solutions which is essential for separation on HILIC column and was efficiently extracted with the same solvent. Moreover, it was relatively separated at the same retention time of the analyte which helps to decrease the separation run time.

Different types of column were tested and Acquity UPLC HILIC BEH 1.7 µm 2.1 × 50 mm showed good separation. HILIC chromatographic separation mechanism depends on physicochemical properties of the stationary phase and mobile phase. The mobile phase for HILIC chromatography includes water-miscible polar organic solvents with a small amount of water or buffer [24]. We attempted several organic solvents to use for the mobile phase. Different strengths of acetate and format buffer concentrations, as well as different pH levels were tested in order to achieve the optimal separation of baricitinib and IS. Due to the nature of acetonitrile as a water-miscible organic solvent with intermediate polarity and lacking an acidic proton characteristic that encourages retention of polar analytes and the ability of ammonium acetate to control pH of the mobile phase and ion strength, they were selected for use as the mobile phase. 20 mM ammonium acetate buffer solution at pH 3 was used for the mobile phase because it helped to suppress baricitinib peak tailing and to obtain symmetric and sharp peaks. Hence, the final choice of the mobile phase was 0.1% formic acid in acetonitrile and 20 mM ammonium acetate buffer (97:3) at pH 3. The flow rate was 0.2 mL/min. The low amount of buffer in the mobile phase is in agreement with Buszewski and Noga [25]. LC-MS/MS method described here showed high sensitivity and has a short run time (3.0 min) which is acceptable for routine analyses, which has advantages over the previous method described by Veeraraghavan et al. [22].

### 2.2. Method Validation

#### 2.2.1. Selectivity and Specificity

**Figure 3:**
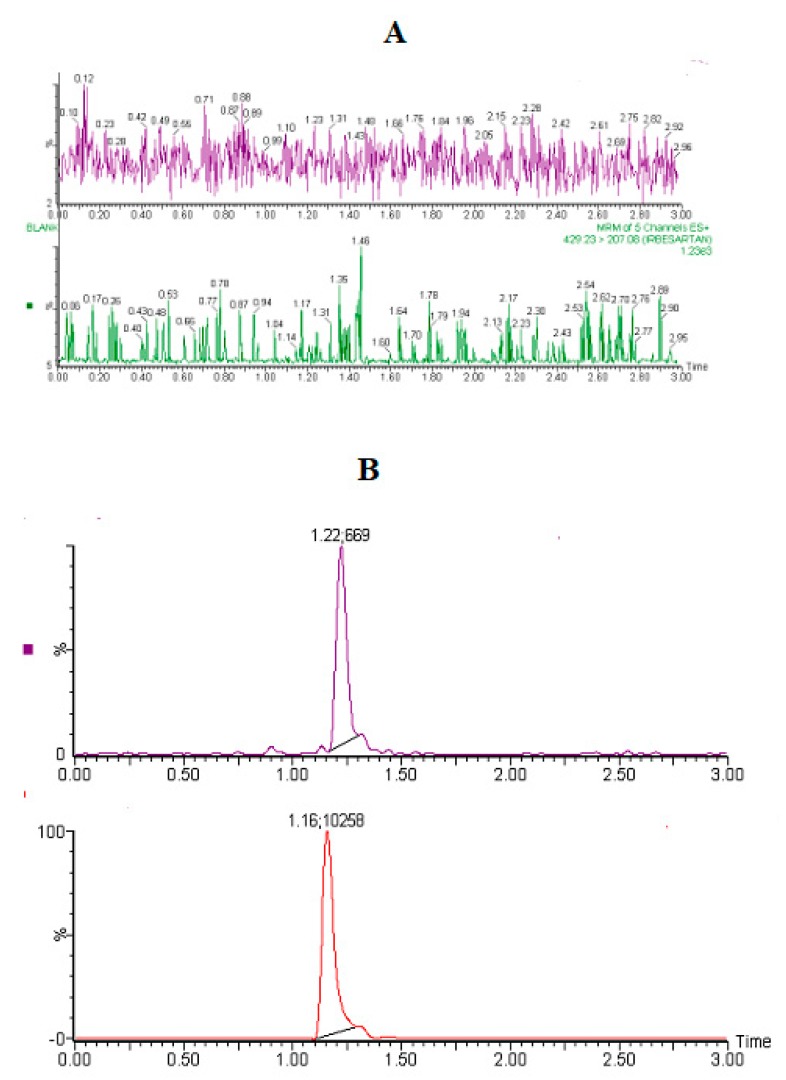
MRM Chromatograms of baricitinib and internal standard in blank rat plasma (**A**), and plasma spiked at LLOQ level (**B**).

Under the optimized conditions, there were no significant interfering peaks from endogenous sources that could be observed at the retention time of baricitinib and IS in the blank plasma obtained from six different rats. The retention times of baricitinib and IS were 1.2 ± 0.02 and 1.16 ± 0.03 min, respectively, with a total run time of 3.0 min only. Additionally, peaks were detected with excellent resolution and good shapes, which concludes acceptable selectivity of the method for routine quantification of baricitinib in plasma samples. Representative MRM chromatograms of the blank plasma did not show any interfering peaks at the elution time of baricitinib and IS, as presented in Figure 3A.

#### 2.2.2. Linearity and Lower Limit of Quantification

The chromatogram of baricitinib and IS in blank plasma and LLOQ are presented in Figure 3B. At the LLOQ, the signal to noise ratio was greater than 5-fold of the response of the blank sample. The calibration curves were constructed by plotting peak area ratios (baricitinib/IS) versus concentrations of baricitinib ranging from 0.2 to 500 ng/mL. The weighing factor of 1/X2 was used for the linear fitting and least-square residual for the calibration curves. The correlation coefficient was found to be ≥0.997, LLOQ was quantified with acceptable accuracy and precision (≤20%) (Table 1). 

#### 2.2.3. Precision and Accuracy

The intra- and inter-day precision and accuracy of the method used are listed in Table 1. Notably, the intra-day and inter-day precision values for the QC concentrations were ≤13.2% and ≤11.4 (expressed as CV %), respectively. Similarly, intra-day and inter-day accuracy ranged from 85.3% to 90.0% and 87.5% to 88.3%, respectively. The results showed that the assay met the desired acceptable precision and accuracy criteria set by regulatory guidelines.

#### 2.2.4. Recovery and Matrix Effects

The extraction efficiency (recovery) and matrix effect of baricitinib using QC samples at three different concentrations (0.6, 40.0, 400.0 ng/mL) and IS (100 ng/mL) are presented in Table 2. The average extraction efficiency of baricitinib was 87.9%. This was consistent and concentration-independent of the CV% value was ≤8.2%. The average matrix effect value of baricitinib between three QC concentration levels was 88.8%. The matrix effect was considered negligible as CV% was ≤10.3% for all QC concentration levels.

#### 2.2.5. Stability

Baricitinib and IS were stable under analysis process and storage conditions. The QC samples analysis showed no significant changes in comparison to nominal concentrations. In all cases, accuracy and precision of QC samples were found to be within the acceptable limits of ±15% against freshly prepared calibration curves as shown in Table 3. Moreover, baricitinib and IS working solutions were stable (with no significant changes) at room temperature (23–25 °C) for 6 h and at refrigerated temperature (2–8 °C) for 10 days.

### 2.3. Application to a Pharmacokinetic Study 

To verify the sensitivity, selectivity, and efficiency of this method, the method was applied in a preliminary pharmacokinetic study of baricitinib in male rats after oral administration of 2 mg/kg suspension of baricitinib. The results of the pharmacokinetic parameters are presented in Table 4. The C_max_ of 129.08 ± 91.4 ng/mL was achieved at 0.5 h after administration of 2 mg/kg of baricitinib suspension. AUC_0–11_ and AUC_0−∞_ were found to be 205.15 ± 101.4 and 222.53 ± 107.2 ng h/mL, respectively. The values of baricitinib pharmacokinetic parameters in the present work are much lower than the values reported by Veeraraghavan et al. [22]. The difference was 5-fold higher C_max_ and 4.3-fold higher AUC. However, the values obtained in the current study is highly comparable to the study reported by Committee for Medicinal Products for Human Use, EMA [26].

**Figure 4:**
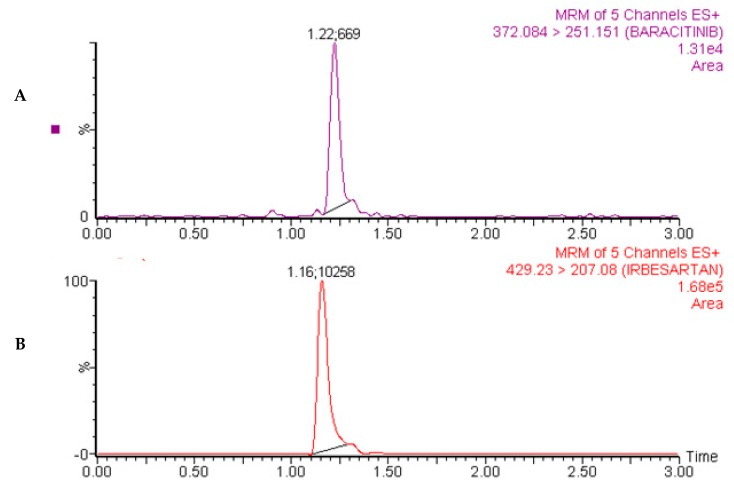
MRM Chromatograms of baricitinib (**A**) and internal standard (**B**) in real rat plasma sample obtained at 1.0 h following oral administration of 2 mg/kg baricitinib.

**Figure 5:**
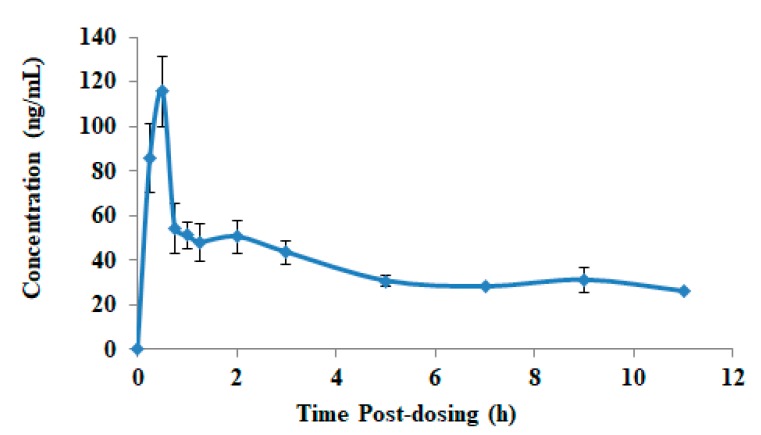
Mean plasma concentration-time profiles of baeacitinib.

Representative MRM chromatograms of baricitinib and IS at 1.0 h after oral administration of baricitinib are shown in Figure 4. Mean plasma concentration versus time profile of baricitinib in rats is also shown in Figure 5. Notably, the LC-MS/MS method satisfied the requirement of routine analyses as it had a short run time (3 min).

The only published method involved protein precipitation procedure with a gradient reversed-phase LC-MS/MS [22]. Although protein precipitation is widely used as a simple and rapid sample preparation technique for bioanalysis, it has some disadvantages. Sample cleaning procedure is relatively poor. The matrix components of extracted samples are not efficiently removed. Therefore, these components may co-elute with the analytes in the isolated supernatant and interfere with the ionization process either by enhancing or suppressing ion production. Moreover, this approach can arise chromatographic separation problems from column fouling or blockage since the efficiency of precipitation is not optimal [27,28]. The present method involved liquid–liquid extraction technique using single step sample extraction procedure. It offers a rapid sample preparation approach without any tedious or time-consuming steps. This method provides efficient peak resolution in isocratic elution mode. There was no need for gradient elution of the analytes as reported by Veeraraghavan et al. [22]. Moreover, the current method is more sensitive and has a wider calibration range than the previously published method. This allows the application of this method not only in pharmacokinetic studies but also in toxicokinetics studies. Moreover, the flow rate of this method is relatively low (0.25 mL/min) which is four times less compared to that of Veeraraghavan et al. [22] method (1.00 mL/min). Furthermore, the run time is short (3 min) with both the analyte and IS eluted at retention time of 1.22 and 1.16 min, respectively. This is shorter run time in comparison with Veeraraghavan et al. method (7.5 min) in which all the analytes of interest eluted between 3.5–5.3 min [22]. Therefore, our method has the advantage of minimizing analysis time, solvent consumption and consequently the relative cost.

## 3. Materials and Methods

### 3.1. Experimental

Baricitinib (>99% purity) was purchased from Enzo Life Sciences, Inc. (Exeter, UK) and irbersartan (Figure 1B) was purchased from Beijing Mesochem Technology Co., Ltd. (Beijing, China). HPLC-grade methanol and acetonitrile were obtained from Avonchem Ltd., (Macclesfield, UK) and Winlab Pty. Ltd. (Brendale, Australia), respectively. Analytical-grade formic acid, dichloromethane, n-hexane, and ammonium acetate were obtained from the BDH Laboratory (Lutterworth, UK). Blank rat plasma was collected and separated from the blood of healthy rats.

### 3.2. Equipment

Waters Acquity TQD UPLC/ Mass spectrometer (Waters Co., Milford, MA, USA) was used in the study. Other equipment used included vortex mixer Taboys^®^ model AP 56 (TROEMNER. Hingham, MA, USA), analytical balance Mettler Toledo^®^ model XS 205 (Greifensee, Switzerland), and a sample concentrator Thermo^®^ Savant SC210A speed Vac (Waltham, MA, USA). Deionized water was prepared by Milli-Q reverse osmosis (Millipore^®^, Bedford, MA, USA).

### 3.3. Chromatographic Conditions

Sample analysis was performed on an Acquity TQD UPLC-MS/MS system (Waters Co., Milford, MA, USA). The triple quadrupole mass spectrometer was operated in the MRM mode, and detection was carried out using electrospray ionization (ESI) in the positive ion mode. In addition, the mass spectrometry parameters including parent to daughter ion transition, collision energy, cone voltage, and dwell time were optimized to determine the analyte and IS (Table 5). The collision gas (argon) flow rate was kept at 0.1 mL/min and the desolvation gas (nitrogen) flow rate was optimized to 600 L/h. Notably, chromatographic separation was achieved using a Waters Acquity UPLC HILIC BEH 1.7 µm 2.1 × 50 mm column with the mobile phase consisting of 0.1% formic acid in acetonitrile and 20 mM ammonium acetate (pH 3). The flow rate was 0.2 mL/min.

### 3.4. Preparation of Stock and Working Solutions

The standard stock solutions of baricitinib and irbesartan were prepared individually by dissolving 5 mg of each drug in dimethyl sulfoxide (DMSO, Loba Chemi, Mumbai, India). Stock solutions were then diluted with methanol to obtain working solutions of 1000 µg/mL for both baricitinib and IS. Further dilution was carried out in acetonitrile to yield working solutions (20 µg/mL) for both the analyte and IS. The calibration range was 0.2 to 500 ng/mL (0.2, 1.0, 5.0, 1.0, 20.0, 100.0, 200.0, and 500.0 ng/mL). Three quality control samples at three concentration levels 0.6, 40.0, and 400.0 ng/mL and lower limit of quantitation (LLOQ; 0.2 ng/mL) were prepared.

### 3.5. Sample Preparation

Baricitinib and IS were extracted from rat plasma using liquid–liquid extraction. 20 µL of irbesartan working solution (20 µg/mL) was added to 100 µL plasma samples. After vortex-mixing for 20 s, 1 mL of *n*-hexane:dichlromethane mixture (1:1) was added. Samples were then mixed for 1 min and centrifuged for 5 min at 5600× *g*. The supernatant (0.8 mL) was transferred to a 5-mL tube and evaporated to dryness at 40 °C. Residues were reconstituted in 100 µL of the mobile phase and 5 µL was injected into the LC-MS/MS system.

### 3.6. Method Validation

The method was validated according to the FDA and EMA guidelines for bioanalytical method validation [29,30].

The selectivity of the proposed method was estimated by analyzing rat blank plasma samples from 6 different sources to detect the interfering peaks at the same retention times of the analyte and IS. The sensitivity of the method is expressed by the LLOQ, which is defined as the lowest concentration of an analyte that can be accurately measured.

The plasma calibration curves were constructed by plotting the peak area ratios (baricitinib/IS) against baricitinib concentrations covering the expected range (0.2–500 ng/mL), including the LLOQ. The correlation coefficient (r^2^) of calibration curves should not less than 0.99. The limit of detection (LOD) is defined as the concentration of an analyte yielding a peak with a signal to noise ratio of 3, while this ratio is 5 times for the low limit of quantification (LOQ).

The accuracy and precision of the method were determined by analyzing three different QC concentrations (low, medium and high QC samples) representing the entire range of the calibration curve and LLOQ samples. Inter-day accuracy and precision were measured consecutively for three days, whereas intra-day accuracy and precision were measured in one day. Accuracy of QC samples should be within ±15% of the nominal concentrations of QC samples and 20% for LLOQ. Precision should not exceed 15% CV% for QC samples and 20% for LLOQ.

Stability of the analyte was assessed using five measurements of QC samples at low and high concentrations after exposure to different conditions of storage and processing temperature. Baracitinib stability was evaluated after three freeze-thaw cycles, storage at room temperature (23–25 °C) for 6 h (short term stability), and subsequent storage for eight weeks at −80 °C (long term stability). Moreover, the stability of stock and working solutions of baricitinib and the IS at room temperature for 6 h (23–25 °C) and at refrigerator (2–8 °C) for 12 days was tested.

The absolute recovery of the method was determined by comparing the average of peak area measurements obtained from the blank plasma spiked before extraction to those obtained from the blank plasma spiked after extraction. The effect of the matrix was evaluated by comparing the peak area ratios of post-extracted plasma spiked with baricitinib and IS to peak area ratio of real solutions contain the same concentration.

### 3.7. Animal

Sprague Dawely rats weighing 200–230 g were obtained from the animal house at the National Organization for Drug Control and Research, Giza, Egypt. Rats were fed standard sufficient food and water and experiments were performed under the Guide for Care and Use of laboratory animals. experimental protocols were approved by the ethics committee of the Faculty of Pharmacy at Al-Azhar University, Cairo, Egypt (no. 206).

### 3.8. Application to a Pharmacokinetic Study

To demonstrate the utility of the present method, a pharmacokinetic study of baricitinib was performed on six male Sprague Dawely rats (200–230 g). Baricitinib was used to create a suspension using 1% carboxymethyl cellulose. Following overnight fasting, the drug was administered in a dose of 2 mg/10 mL/kg. Blood samples (approximately 0.25 mL) were then collected from the retro-orbital plexus into heparinized tubes at 0.0, 0.25, 0.5, 0.75, 1.0, 1.25, 2.0, 3.0, 5.0, 7.0, 9.0, and 11.0 h post-dosing. Plasma samples were obtained by centrifuging the blood at 4250× *g* for 5 min and stored frozen at −80 °C until analysis. The pharmacokinetic parameters C_max_, t_max_, AUC, t_½_, and K_el_ were calculated using WinNonlin software.

## 4. Conclusions

A new rapid, sensitive, selective, reproducible, precise, and accurate UPLC-MS/MS method was developed and validated for the baricitinib plasma quantitation of. The method met the acceptance criteria for bioanalytical method validation defined by the recent FDA and EMA guidelines. The method was successfully applied in pharmacokinetic study following oral administration of a single dose of baricitinib in male rats under fasting conditions. This method demonstrates its applicability in relevant preclinical, therapeutic drug monitoring and potentially for pharmacokinetic studies of the baricitinib-drug interaction.

## Figures and Tables

**Table 1:** Intra-day and inter-day precision and accuracy values of baracitinib in rat plasma.

Nominal Conc. (ng/mL)	Intra-Day			Inter-Day		
	Measured Conc. (ng/mL)	CV (%)	Accuracy (%)	Measured Conc. (ng/mL)	CV (%)	Accuracy (%)
0.2	0.17 ± 0.02	13.2	85.3	0.175 ± 0.02	11.4	87.5
0.6	0.54 ± 0.06	11.8	89.4	0.53 ± 0.06	11.3	88.3
40.0	35.99 ± 4.00	11.1	90.0	35.24 ± 3.30	9.4	88.1
400.0	344.36 ± 2.49	2.8	86.9	353.16 ± 6.61	1.9	88.3

**Table 2:** Recovery and matrix effect of baracitinib and IS in rat plasma (*n* = 6).

Drug Name	Nominal Conc. (ng/mL)	Extraction Recovery			Matrix Effects		
		Mean ± SD	Accuracy (%)	CV (%)	Mean ± SD	Accuracy (%)	CV (%)
Baracitinib	0.6	0.58 ± 0.04	95.9	5.4	0.63 ± 0.04	89.3	6.8
	40.0	34.37 ± 1.85	85.9	8.2	0.53 ± 0.04	89.5	6.8
	400.0	351.67±31.32	81.7	0.7	349.54 ± 35.94	87.7	10.3
Irbersartan	100.0	74.33 ± 3.21	74.3	4.3	87.40 ± 2.95	87.4	3.5

**Table 3:** Stability quality control sample of baracitinib in rat plasma (*n* = 6).

Stability	Conc. (ng/mL)					
	40.0 ng/mL			400.0 ng/mL		
Parameters	Mean ± SD	Accuracy (%)	Precision (%CV)	Mean ± SD	Accuracy (%)	Precision (%CV)
Bench top (6 h)	34.61 ± 3.03	8.7	86.5	343.27 ± 44.76	85.8	13.0
Thaw/freeze (3 cycles)	34.09 ± 3.99	85.1	8.8	345.73 ± 39.84	86.4	11.5
Auto-sampler	35.6 ± 2.69	89.0	7.6	360.36 ± 11.52	90.1	11.5
Long term (at −80 °C for 8 weeks)	34.63 ± 4.63	86.6	13.4	350.56 ± 28.53	87.64	8.1

**Table 4:** Pharmacokinetic Parameters (Mean ± SD) of baracitinib Following Administration of 2 mg/kg to rats.

Parameters	Mean * ± SD
C_max_ (ng/mL)	129.08 ± 91.4
AUC_0-11_ (ng.h/mL)	205.15 ± 101.40
AUC_0-inf_ (ng.h/mL)	222.53 ± 107.20
K_el_ (h)	0.32 ± 0.04
t_1/2_ (h)	2.24 ± 0.43
MRT (h)	3.30 ± 0.78
t _max_ (h)	0.5

*: Median for t_max._ C_max_, maximum concentration; T_max_, time to reach maximum concentration; AUC_0-11_, area under the concentration time curve from 0 to 11 h; AUC_0-inf_, AUC from 0 h to infinity; t_1/2_, half-life; MRT, median residence time.

**Table 5:** Mass optimization parameter for, baracitinib and irbersartan (IS).

Parameters	Baracitinib	Irbersartan
I. Parameters of compound-dependent		
SRM transition (m/z) (Parent)	372.15	429.20
Daughter	251.24	207.35
Collision energy (eV)	52	38
Cone voltages	30	22
II. Parameters of source-dependent		
Collision gas	Argon with a flow rate of 0.1 mL/min	
Desolvating gas	Nitrogen with flow rate of 600 L/h	
Desolvating temperature (°C)	350	
Source temperature was (°C)	150	
The capillary voltage (kV)	4	
